# Conformal Retinal Image Sensor Based on Electrochemically Exfoliated MoS_2_ Nanosheets

**DOI:** 10.3390/nano15080622

**Published:** 2025-04-18

**Authors:** Tianxiang Li, Hao Yuan, Wentong Cai, Qi Su, Lingxian Kong, Bo Sun, Tielin Shi

**Affiliations:** 1 Aviation Key Lab of Science and Technology on High Performance Electromagnetic Windows, Jinan 250023, China; litianxiang996@163.com (T.L.); yuanhao178163@163.com (H.Y.); efficientc@126.com (W.C.); 2 Innovation Center for Electromagnetic Functional Structure, Jinan 250023, China; 3 School of Aerospace Engineering, Huazhong University of Science and Technology, Wuhan 430074, China; qisu@hust.edu.cn (Q.S.); bosun@hust.edu.cn (B.S.); 4 State Key Laboratory of Intelligent Manufacturing Equipment and Technology, Huazhong University of Science and Technology, Wuhan 430074, China; tlshi@hust.edu.cn; 5 Shenzhen Huazhong University of Science and Technology Research Institute, Shenzhen 518057, China

**Keywords:** electrochemical exfoliation, MoS_2_ photodetector, retinal imaging, spherical conformal surface

## Abstract

Retina-like photoimaging devices with features such as a wide-field-of-view and high spatial resolution have wide application prospects in retinal prosthetics and remote sensing. However, the fabrication of flexible and conformal surfaces is hindered by the incompatible microfabrication processes of traditional rigid, silicon-based substrates. A kirigami strategy for hemispherical surface assembly is proposed to construct a MoS_2_-based retina-like photodetector array. The device is first fabricated on a flat polyimide (PI) substrate and then tailored using a laser. By approximating the spherical surface using planar sectors, the laser-cut PI film can tightly adhere to the PDMS spherical shell without significant wrinkles. The responsivity and specific detectivity of our conformal photodetector can reach as high as 247.9 A/W and 6.16 × 10^11^ Jones, respectively. The array integrates 180 pixels on a spherical crown with a radius of 11 mm, and a hollow letter “T” is successfully recognized. Comprehensive experimental results in this work reveal the utility of our device for photoelectric detection and imaging. We believe that our work provides a new methodology for the exploitation of 2D material-based retinal image sensors.

## 1. Introduction

Soft image sensors with retinal imaging functions are in high demand in artificial visual systems, such as robotics, night surveillance, and medical imaging [1,2,3], which have a wide-field-of-view and no aberration. Traditional flat Si-based photodetector arrays need a complex optical lens system to eliminate aberrations and ensure that light can be focused on the focal plane [4,5,6]. However, integrating photodetectors on retinal concave surfaces is challenging due to the incompatible planar wafer fabrication industry [7,8]. Additional restrictions, such as mechanical rigidity, further hinder the use of Si in wearable and implantable retinal imaging systems. Therefore, novel device designs and new sensitive materials are urgently required for conformal retinal image sensors. The simplest approach to fabricating imaging units on a retinal surface is to integrate all sensors on a plane substrate and then transfer them onto a hemispherical concave, which necessitates that the substrates and photosensitive materials be flexible and stretchable [9].

Recently, several hemispherical image sensors have been proposed for this purpose. Lee et al. proposed a hemispherical organic dye-sensitized graphene photodetector array by transferring fractal web PI supporting layers onto a PDMS dome [10]. However, the different sizes of PI and PDMS created wrinkles at the edges of the fractal webs. Hu et al. fabricated a 5 × 5 In/MoS_2_ synaptic imaging array on a 20 mm rigid hemispherical quartz substrate, which can achieve image sensing and memory with extremely low energy consumption [11]. Despite the progress mentioned above, problems such as low resolution and nonconformal substrates still exist. To promote the use of retinal image sensors in artificial vision systems, it is necessary to effectively assemble optoelectronic devices onto non-planar surfaces [12,13]. Recently, kirigami has been proposed as a conformal strategy to solve the problem of non-stretchable substrates conforming to curved surfaces [14,15]. Precise kirigami inspired us to assemble 2D sheets on various 3D surfaces.

Over the past few decades, two-dimensional (2D) materials, such as MoS_2,_ have emerged as a transformative platform for flexible electronic devices owing to their unique optoelectronic properties and mechanical flexibility [16,17,18]. Atomically thin MoS_2_ exhibits exceptional properties, including strong resonant light absorption (>20%), layer-dependent bandgap, and mechanical flexibility (Young’s modulus of ~270 GPa), making it an ideal photosensitive material for flexible image sensors [19,20]. Nevertheless, the scalable fabrication of large-area, high-quality MoS_2_ films with uniform photoresponsivity and low defect density remains a critical bottleneck, particularly for retinal imaging applications. Recent advances in electrochemical intercalation strategies for 2D material crystals using quaternary ammonium ions have provided promising routes for engineering wafer-scale MoS_2_ films [21,22,23].

In this study, we demonstrate a retinal image sensor using a tailored and spliced PI substrate with photosensitive materials made of spin-coated MoS_2_ nanosheets. Defect-free MoS_2_ nanosheets are prepared by electrochemical intercalation coupled with mild sonication. Moreover, the intercalation current and microtopography of the intercalated crystals are closely monitored throughout the entire intercalation process to ensure the adequacy of intercalation to obtain high-quality and few-layer MoS_2_ nanosheets. The imaging device is fabricated by spin-coating MoS_2_ nanosheets onto PI/Si substrates with electrodes and cutting them according to kirigami graphics using a laser. The carefully designed pattern ensures that the PI films can be perfectly spliced on spherical concave surfaces without any wrinkles. The device has a wide-field-of-view of 150° and integrates 180 pixels on a spherical crown with a radius of 11 mm, which demonstrates great potential for artificial visual systems. This study paves the way for a universal route to fully exploit integrated conformal retinal image sensors based on 2D materials.

## 2. Method

### 2.1. Electrochemical Exfoliation of MoS_2_ Crystal

We prepared MoS_2_ nanosheet dispersion using an electrochemical exfoliation method [21]. The electrochemical exfoliation of MoS_2_ crystals is schematically illustrated in Figure S1. The intercalation process was implemented in a beaker, and a 10 mg/mL solution of teraheptylammonium bromide (THAB) (98%, TCI) in acetonitrile was used as the intercalation electrolyte. A MoS_2_ crystal and a graphite rod served as the cathode and anode, respectively, in the two-electrode setup. MoS_2_ was clipped with a flat-nose alligator clip and submerged in the electrolyte. The intercalation process continued for 2 h at a voltage of 10 V DC, leading to a significant expansion of the volume of the MoS_2_ crystal (Figure S2). In the cathode, MoS_2_ was exposed to a negative electrochemical potential, and a certain number of THA^+^ cations were intercalated into the MoS_2_ host crystal, forming a (THA^+^)*_x_*MoS_2_*^x^*^−^ compound. Afterward, the compounds were decomposed to regenerate MoS_2_. At the same time, the bromide ions were oxidized at the anode. The reaction can be expressed as follows [22,24]:

Cathode: MoS_2_ + *x*THA^+^ + *x*e^−^ → (THA^+^)*_x_*MoS_2_*^x^*^−^

(THA^+^)*_x_*MoS_2_*^x^*^−^ → MoS_2_ + trialkylamines + alkenes

Anode: *x*Br^−^ → (*x*/2) Br_2_ + *x*e^−^

The intercalated bulk appeared soft and fluffy, making it convenient to shred into smaller pieces and sonicate in 40 mL of 0.2 M PVP/DMF solution for 1 h to obtain the MoS_2_ dispersion. Differential centrifugation was used to separate a few-layer MoS_2_ from the dispersion. The dispersion was first centrifuged at 3000 rpm for 5 min to separate the thick layers of MoS_2_, and the supernatant solution was centrifuged again at 9000 rpm for 15 min. The sediment was dispersed in isopropanol and centrifuged again, with this process repeated five times to ensure that the surfactant PVP was sufficiently washed away, and a homogeneous dispersion of pure 2H-phase MoS_2_ nanosheets was obtained.

### 2.2. Spherical Surface Disassembly and Device Fabrication

An approximate projection is utilized to reduce the dimensions of the spherical surface from 3D to 2D. We divide the spherical surface into 12 equal parts according to the central angle. Because every part is identical, we will only discuss one of those sections, where the pole becomes the coordinate origin in the Cartesian coordination system, and the projection of the central meridian becomes the x-axis. The mapping relationship between the point on the spherical surface P_s_ (R, θ) and the projection point on the plane coordination P_p_ (x, y) is as follows:

x=Rθ

$$y=\frac{\pi }{n}Rsin\theta$$

where R and θ are the radius of the sphere and the longitude of the point. Then the relationship between x and y can be expressed as:

$$y=\frac{\pi }{n}Rsin\frac{x}{R}$$

The perspective view of the kirigami process is plotted in Figure S3.

## 3. Result and Discussion

**Figure 1:**
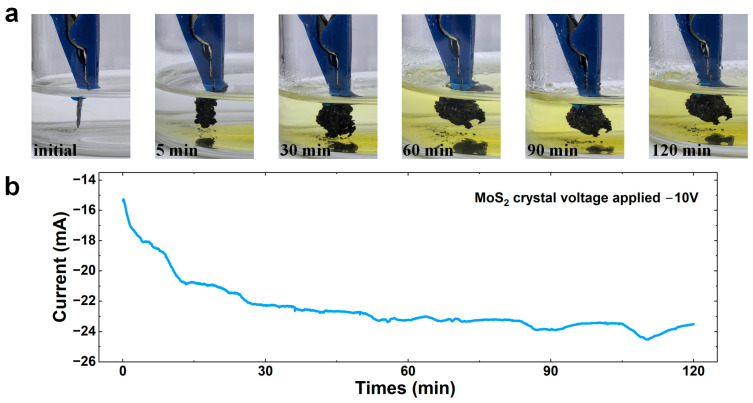
(**a**) Morphology evolution of MoS_2_ crystal after 0, 5 min, 30 min, 60 min, 90 min, and 120 min intercalation, respectively. (**b**) Intercalation current curve between the MoS_2_ working electrode and the graphite electrode as a function of time.

Figure 1a illustrates the volume transformation of the MoS_2_ crystal during the electrochemical intercalation process. During this process, the crystal undergoes expansion along the direction perpendicular to the layers as the THA^+^ ions are intercalated into the layers. Simultaneous to the expansion, the solution surrounding the graphite rods turns yellowish, indicating the generation of bromine at the anode [24]. An electrochemical workstation was utilized to supply a constant voltage of 10 V to the electrolytic cell and record the intercalation current throughout the entire process. Based on the optical microscope images (Figure 1a) and current curves (Figure 1b), the intercalation process can be divided into two distinct stages. From 0 to 30 min (stage I), the MoS_2_ volume expands rapidly, driving the crystal closer to the graphite electrode, which subsequently leads to a continuous increase in the intercalation current. From 30 to 120 min (stage II), the intercalation process proceeds steadily, although the occasional shedding of fragments from the intercalated crystal surface can cause fluctuations in the current curve [25].

**Figure 2:**
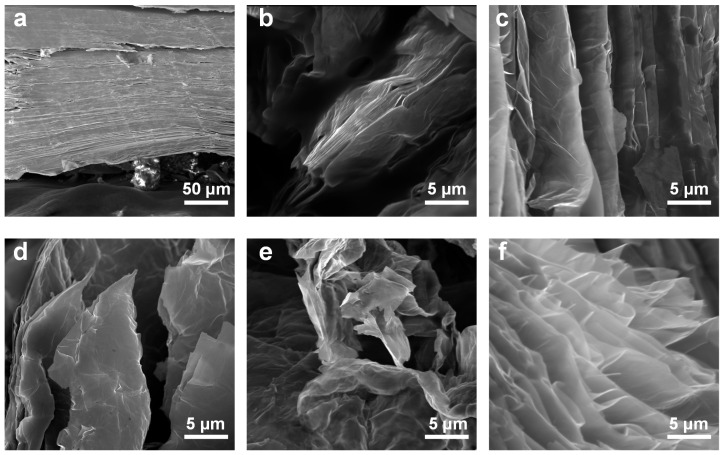
Comparing the SEM photographs of the MoS_2_ crystal with varying degrees of intercalation. (**a**) The SEM micrograph of the MoS_2_ crystal before electrochemical intercalation. (**b**–**f**) SEM micrographs of MoS_2_ crystal after electrochemical intercalation for 5, 30, 60, 90, and 120 min, respectively.

In Figure 2, ex-situ SEM images were collected to further elucidate the intercalation process. As shown in Figure 2a, the cross-section of the MoS_2_ crystal without electrochemical intercalation is composed of tightly stacked lamellar structures. After 5 min of intercalation, the crystal exhibits significant swelling with THA^+^ ions, leading to pronounced separation along the interlayers and forming ridge-like thick structures (Figure 2b). When the intercalation time is prolonged to 30 min (Figure 2c), the ridge-like thick structures further transform into an accordion-like thin multi-layered structure. Although the macroscopic morphology of the expanded MoS_2_ crystal no longer changes after 30 min of intercalation, intercalation continues to occur at a tiny level, as shown in Figure 2d–f. Under the driving of the electric field force, THA+ ions intercalated into the interlayers of the MoS_2_ crystal, forming a compound (THA^+^)*_x_*MoS_2_*^x^*^−^. However, the intercalated compound decomposes rapidly, which contributes to further increasing the interlayer distance of MoS_2_ [22,24]. In conclusion, we gain a comprehensive understanding of the intercalation process through photographs, current measurements, and SEM. Despite the crystal volume expanding to its maximum, the desired thin layer structures have not yet been fully achieved, indicating that further intercalation is required to ensure that the reaction proceeds sufficiently.

**Figure 3:**
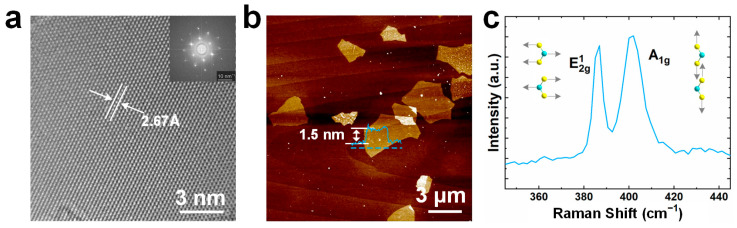
(**a**) HRTEM image of exfoliated MoS_2_ nanosheets (Insert is FFT pattern of the corresponding area). (**b**) AFM image of exfoliated MoS_2_ nanosheets with a thickness of 1.5 nm. The blue line represents the height profile across the blue dashed line, indicating a thickness of 1.5 nm. (**c**) Raman spectroscopy of exfoliated MoS_2_ nanosheets, showing Raman vibration of E_2g_^1^ mode and A_1g_ mode.

To investigate the crystal structure of the exfoliated MoS_2_ nanosheets, the dispersion was dropped onto a copper grid for the TEM test. A uniform single hexagonal lattice structure can be observed in the high-resolution transmission electron microscopy (HRTEM) image (Figure 3a), from which the d-spacing was measured to be 0.267 nm, corresponding to the interplanar spacing of the (100) planes. In addition, the fast Fourier transform (FFT) pattern in the insert of Figure 2d shows distinct hexagonal diffraction spots of the (100) and (110) planes conforming to the 2H lattice structure of exfoliated MoS_2_ nanosheets [26]. Atomic force microscopy (AFM) was employed to determine the thickness of the exfoliated MoS_2_ nanosheets, as shown in Figure 3b. The height of the cross-sectional profile of the exfoliated nanosheet is 1.5 nm, indicating that the nanosheet is a bilayer. The Raman spectrum of the nanosheet on the Si substrate is presented in Figure 3c. The peaks located at 385.5 cm^−1^ and 404.3 cm^−1^ are observed to be the E_2g_^1^ mode and the A_1g_ mode, revealing the spacing of both peaks of 18.8 cm^−1^, corresponding to the in-plane vibration mode of opposite Mo-S atoms and out-of-plane vibration of S atoms, respectively [27,28,29]. The Raman shift between E_2g_^1^ and A_1g_ indicates the few-layer structure of the MoS_2_ nanosheets.

**Figure 4:**
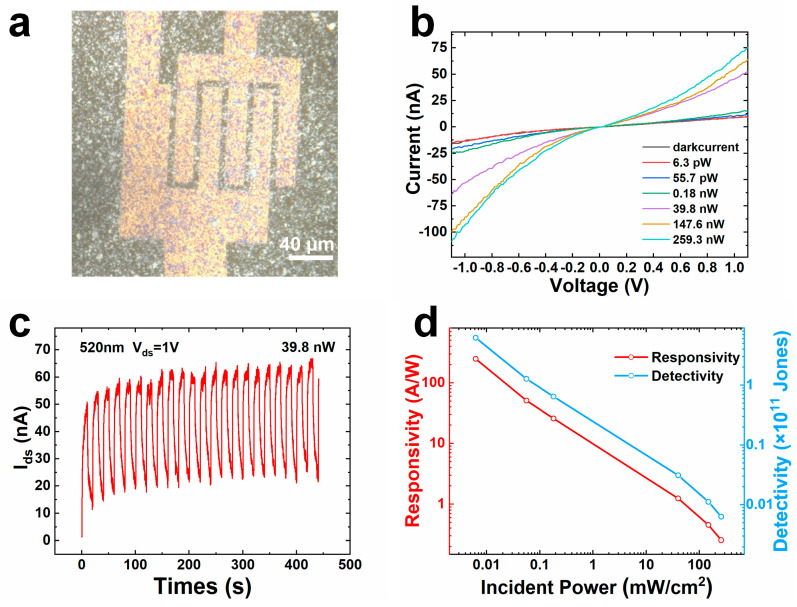
(**a**) Optical microscope image of a spin-coated MoS_2_ photodetector in the retinal photodetector array (the scale bar is 20 μm). (**b**) I–V characteristics of the photodetector under dark and illumination with 6.3 pW, 55.7 pW, 0.18 nW, 39.8 nW, 147.6 nW, and 259.3 nW, respectively. (**c**) The transient photoresponse under illumination with a 20 s switching period at 39.8 nW. (**d**) The R and D* as functions of illumination power.

We subsequently fabricated the device by standard photolithography and spin-coating processes on a 2-inch wafer. The design of the mask in photolithography and the device on the wafer are shown in Figure S4. After transferring the device to a conformal spherical concave, the performance of a single pixel in the array was systematically investigated under 520 nm laser illumination. A local photograph of one photodetector in the array is recorded using an optical microscope, as shown in Figure 4a, in which the channel width and effective sensitive area are 5 μm and 2400 μm^2^, respectively. Figure 4b shows the I–V characteristics of the photodetector in the dark and under illumination with different powers. The Schottky barrier existing between MoS_2_ nanosheets and Au electrodes makes the I-V curves nonlinear [30,31]. The photocurrent increases from 1.6 nA to 66 nA as the effective incident power changes from 6.3 pW to 259.3 nW, as shown in Figure S5. The relationship between the photocurrent and incident power can be described by Equation (S1), and *λ* was calculated to be 0.38 [32,33]. To further evaluate the optical characteristics of the spin-coated device, the time-resolved photoresponse was recorded at a 1 V bias, as shown in Figure 4c. The switching period was set to 20 s, and the illumination power was 39.8 nW. From the transient response plot, it can be observed that the dark current is initially around 1.6 nA before the laser is turned on, which is consistent with the I–V curves. The current exhibits a sudden increase to about 50 nA at the moment of the laser activation, while the current only decays to around 15 nA after 10 s of laser off, which is significantly higher than the dark current. The switching period response curve in Figure 4c is extracted from Figure S6 to obtain the response time. The rise time and decay time are 3.12 s and 8.07 s, respectively. The prolonged decay time may result from the defects existing at the edges of the stacked nanosheets. Responsivity (R) and specific detectivity (D*) are two important parameters for evaluating the photodetection performance of a photodetector. R is defined by the photocurrent generated from the effective unit incident power on the effective area and can be calculated using Equation (S2) [34,35]. D* represents the ability of the photodetector to detect weak light signals and can be calculated using Equation (S3) [36,37].

As important parameters, the noise equivalent power (NEP) and external quantum efficiency (EQE) are calculated using Equation (S4) and Equation (S5), respectively. The minimum NEP and maximum EQE are 6.45 × 10^−12^ WHz^−1/2^ and 592.6 under the incident power of 6.3 pW, respectively. In addition, R and D* are illustrated as functions of the incident power in Figure 4d. During the process of increasing the incident power, the photo response continuously decreases, which can be attributed to the higher photoconductive gain (G) at lower incident powers. G is directly proportional to the ratio of the hole recombination lifetime (τ_p_) to the transit time (τ_t_). Holes are more likely to be captured by traps at a lower incident power. Trapped holes must first be excited back into the valence band and then recombine with electrons. This process significantly prolongs τ_p_, thereby enhancing the G of the device, which corresponds to higher R and D* at lower incident powers [38,39,40]. While the carrier concentration rises significantly at higher incident power, this leads to an increased probability of recombination between carriers, which reduces the number of free carriers and causes the photocurrent to approach saturation. As a result, the responsivity (R) and detectivity (D*) are lower at high incident powers. The maximums of R and D* are obtained under 6.3 pW effective power irradiation, which can reach as high as 247.9 A/W and 6.16 × 10^11^ Jones, respectively, which is superior to many research reported so far (Table S1) [41,42,43,44,45,46,47].

**Figure 5:**
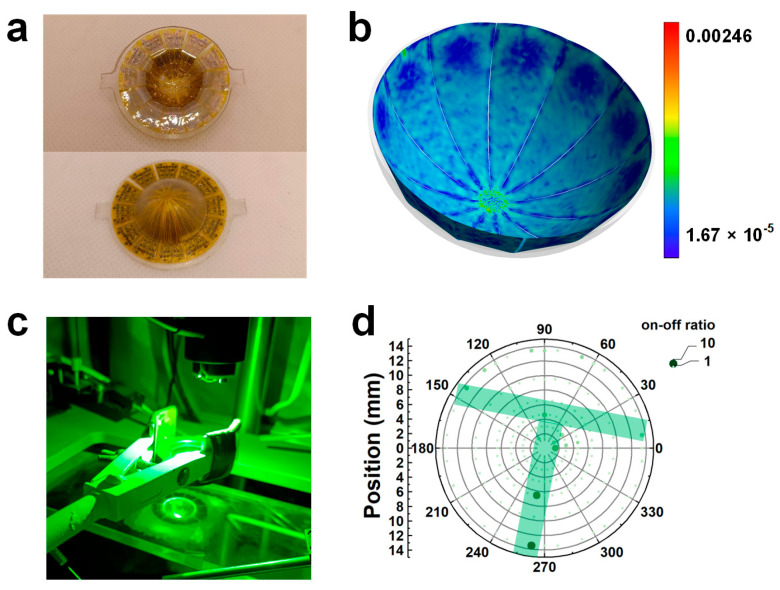
(**a**) Photograph of the conformal retinal image sensor (the upper one is a photograph of the concave surface, and the lower one is a photograph of the convex surface). (**b**) Strain distribution of tailored PI film adhering to a hemispherical concave surface by finite element simulation. (**c**) Photograph of the imaging test setup. (**d**) Mapping projection in a 2D plane of on-off ratio using a T-shaped hollow mask.

The conformal retinal image sensor, which consists of 180 photodetector units, is shown in Figure 5a. The upper image is a photograph of the concave surface, and the lower image is a photograph of the convex surface. The radius of the spherical shell is 11 mm, and the field angle is designed to be 150 degrees, which is similar to that of the human eyes. We conducted a finite element analysis of the process of tailored PI sheets adhering to a hemispherical concave surface. The stress distribution in the spliced PI sheets, as depicted in Figure S7, shows that the maximum stress is about 6.1 MPa. The strain distribution shown in Figure 5b is calculated using ε = σ/E (Hooke’s Law), where ε, σ, and E are the strain, stress, and Young’s modulus, respectively. Given that the Young’s modulus of PI is 2.48 GPa, the maximum strain in the PI sheets is approximately 0.246%, which is significantly less than the yield strain of PI (around 10%). Our meticulous design allows the PI substrate carrying the imaging array to tightly adhere to the concave surface of the spherical substrate without causing warping or bubbles.

Figure 5c shows a photograph of the measurement setup for the conformal retinal image sensor. The laser passes through a hollow mask and a planoconvex lens and then strikes the concave surface of the image sensor. The hollow pattern in the mask is the letter “T”. Figure 5d shows the photoimaging result projected onto the horizontal plane based on the on-off ratio mapping at a 1 V bias. Although the imaging performance suffers from a non-uniform nanosheet distribution and damaged pixels, the shape of the letter “T” can be distinguished, as highlighted by the shadow in Figure 5d. The conformal image sensor has significant potential for wide-field-of-view and high-resolution retina-like imaging.

## 4. Summary

This work presents a remarkable conformal retina-like image sensor based on electrochemically exfoliated MoS_2_ nanosheet channels and a kirigami strategy on a jointed spherical substrate. We have revealed the entire process of electrochemical intercalation through a series of characterizations, including photographs, current measurements, and SEM. High-quality MoS_2_ nanosheets obtained by precise electrochemical exfoliation ensured the excellent performance (responsivity >240 A/W) of the photodetector. By dismantling the 3D spherical surface into 2D sheets, we first fabricated the device on a planer PI substrate. The cutting patterns can be joined into spherical concave conformally and seamlessly, which offers a method for designing spherical image sensors with high precision. In addition, a conformal retina-like image sensor was used to detect a T-shaped hollow mask, which is promising for artificial visual systems. The design of dimension reduction provides new opportunities for the fabrication of spherical optoelectronic devices based on 2D materials.

## Data Availability

Data are contained within the article and Supplementary Materials.

## Supplementary Materials

The following supporting information can be downloaded at: https://www.mdpi.com/article/10.3390/nano15080622/s1, Figure S1. Schematic of electrochemical exfoliation of MoS_2_ bulk to obtain MoS2 nanosheet ink, including electrochemical intercalation, ultrasonication, centrifugation, and redispersion. Figure S2. (a) Photography of the experimental setup. (b) Before electrochemical intercalation of a MoS_2_ crystal. (c) After electrochemical intercalation of a MoS_2_ crystal. Figure S3. (a) Schematic illustration of dismantling 3D spherical surface to 2D sheets. (b) Coordinate transformation from spherical surface to plane coordination. (c) Schematic illustration of tailored 2D sheets by kirigami. Figure S4. (a) The mask pattern design in plane of conformal retinal sensor. (b) Photograph of the device on a 2-inch wafer. Figure S5. The photocurrent as functions of illumination power. Figure S6. The rise time and decay time of our device. Figure S7. The stress distribution of PI films after adhering to a concave spherical surface calculated by finite elements simulation. Table S1. Solution-processable 2D materials photodetectors performances comparison of this work with previous works.
