# Supplementary material for: Conformal Retinal Image Sensor Based on Electrochemically Exfoliated MoS2 Nanosheets
Source: Nanomaterials (Basel). 2025 Apr 18;15(8):622. doi: 10.3390/nano15080622 (PMC12029796; doi:10.3390/nano15080622)
Supplement: Supplementary file 1 [file nanomaterials-15-00622-s001.zip › nanomaterials-3546812-supplementary.pdf]

Conformal Retinal Image Sensor Based on Electrochemically Exfoliated MoS_2 Nanosheets

Tianxiang Li [1,2], Hao Yuan [1,2], Wentong Cai [1,2], Qi Su [3], Lingxian Kong [4,*], Bo Sun [3,5] and Tielin Shi [4]

[1]Aviation Key Lab of Science and Technology on High Performance Electromagnetic Windows, Jinan 250023, China

[2]Innovation Center for Electromagnetic Functional Structure, Jinan 250023, China

[3]School of Aerospace Engineering, Huazhong University of Science and Technology, Wuhan 430074, China

[4]State Key Laboratory of Intelligent Manufacturing Equipment and Technology, Huazhong University of Science and Technology, Wuhan 430074, China

[5]Shenzhen Huazhong University of Science and Technology Research Institute, Shenzhen 518057, China

*Correspondence: lingxiankong@hust.edu.cn

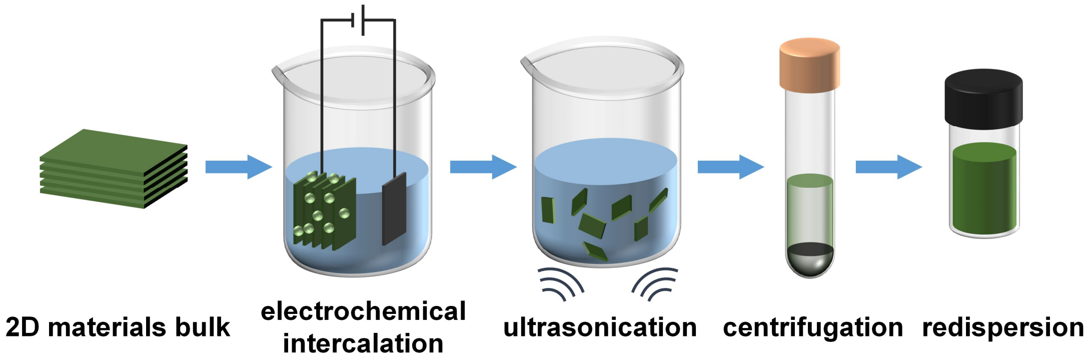

Figure S1. Schematic of electrochemical exfoliation of MoS_2 bulk to obtain MoS2 nanosheet ink, including electrochemical intercalation, ultrasonication, centrifugation, and redispersion.

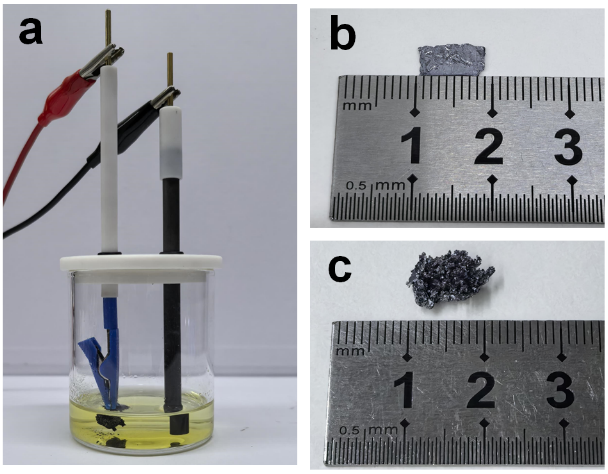

Figure S2. (a) Photography of the experimental setup. (b) Before electrochemical intercalation of a MoS_2 crystal. (c) After electrochemical intercalation of a MoS_2 crystal.

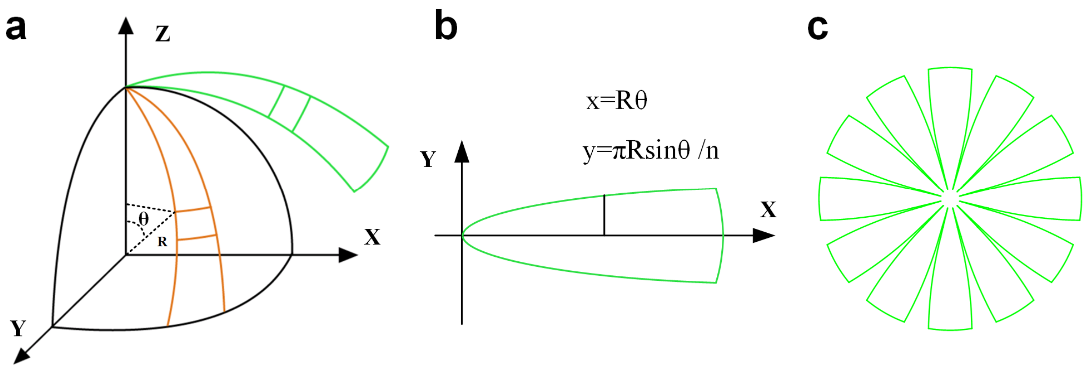

Figure S3. (a) Schematic illustration of dismantling 3D spherical surface to 2D sheets. (b) Coordinate transformation from spherical surface to plane coordination. (c) Schematic illustration of tailored 2D sheets by kirigami.

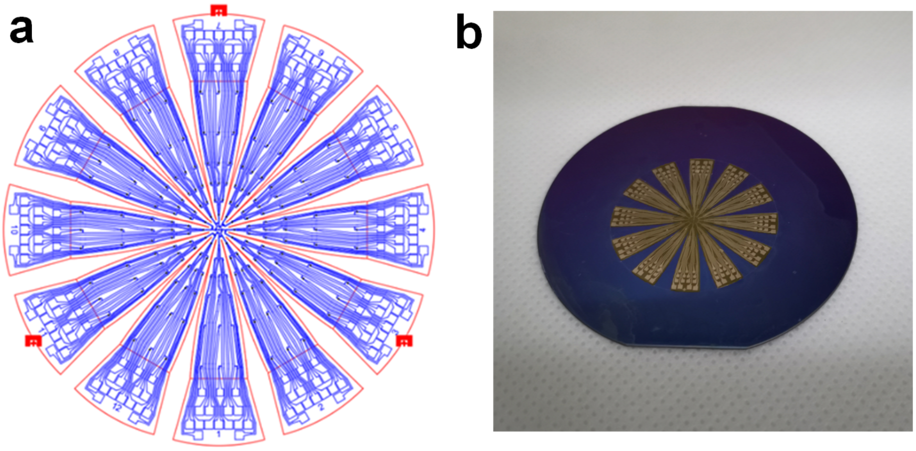

Figure S4. (a) The mask pattern design in plane of conformal retinal sensor. (b) Photograph of the device on a 2-inch wafer.

The relationship between photocurrent and incident power can be described by:

$$I_{ph} \propto P^{\lambda} \qquad \text{(Eq. S1)}$$

Where λ is fitting parameter.

R is defined by the photocurrent generated from per effective unit incident power on the effective area and can be expressed as:

$$\mathrm{R} = \frac{I_{ill} - I_{dark}}{P_{in}} \qquad \text{(Eq. S2)}$$

where the I_{ill}, I_{dark}, and P_{in} are the current under illumination, dark current, and effective incident power, respectively.

D* represents the ability of the photodetector to detect weak light signals and can be defined as:

$$\mathrm{D}^{*} = \frac{\sqrt{A_d}}{NEP} \qquad \text{(Eq. S3)}$$

where A_d and NEP are effective area and noise equivalent power, respectively.

At 1V bias, the noise of the detector mainly originates from the shot noise induced by the dark current. The NEP can be obtained as:

$$NEP = \frac{\sqrt{i_n^2}}{R} = \frac{\sqrt{2qI_{dark}}}{R} \qquad (Eq. S4)$$

where i_n, q, I_{dark} and R are total noise current, electron charge, dark current, and responsivity of the photodetector, respectively.

The external quantum efficiency (EQE) can be obtained by:

$$\mathrm{EQE} = \mathrm{R}\frac{hc}{q\lambda} \qquad (Eq. S5)$$

where h is Planck's constant, c is velocity of light, and q is electron charge. R and λ are responsivity and incident light wavelength, respectively.

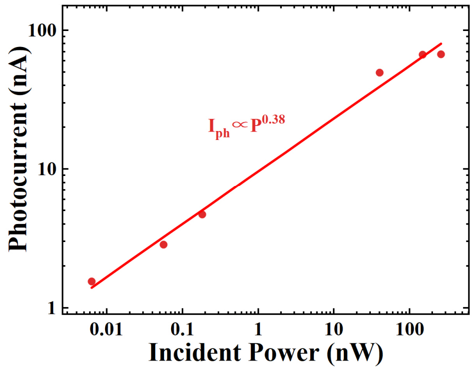

Figure S5. The photocurrent as functions of illumination power.

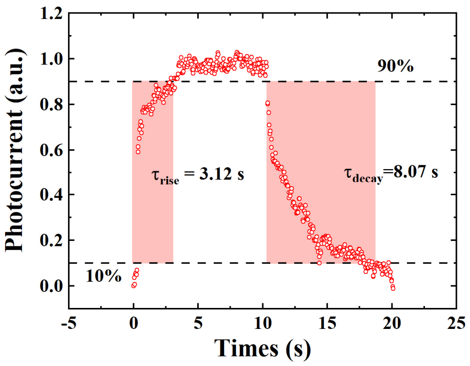

Figure S6. The rise time and decay time of our device.

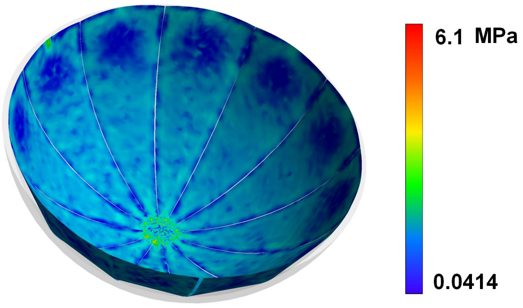

Figure S7. The stress distribution of PI films after adhering to a concave spherical surface calculated by finite elements simulation.

Table S1. Solution-processable 2D materials photodetectors performances comparison of this work with previous works.

Method	R	D*	References
Electrochemical exfoliation	247.9 A/W	6.16×10^{11} Jones	This work
Liquid phase exfoliation	1.75 μA/W	0.5×10^{8} Jones	[S1]
Liquid phase exfoliation	2.7×10^{3} A/W	1.8×10^{7} Jones	[S2]
Liquid phase exfoliation	15 mA/W	2.83×10^{9} Jones	[S3]
Liquid phase exfoliation	27.81 mA W^{-1}	3.96×10^{10} Jones	[S4]
Liquid phase exfoliation	1.7 A/W	--	[S5]
Liquid phase exfoliation	--	~1.8×10^{8} Jones	[S6]
Liquid phase exfoliation	1.08 mA/W	--	[S7]

References:

S1. Chauhan, B.L.; Bhakhar, S.A.; Pataniya, P.M.; Gupta, S.U.; Solanki, G.K.; Pathak, V.M.; Patel, V., Liquid-Phase Exfoliation of WSe2 Nanosheets for ITO/WSe2 Photodetector. *Journal of Materials Science: Materials in Electronics* **2022**, *33*, (13), 10314-10322.

S2. Kuo, L.; Sangwan, V.K.; Rangnekar, S.V.; Chu, T.C.; Lam, D.; Zhu, Z.; Richter, L.J.; Li, R.; Szydłowska, B.M.; Downing, J.R.; Luijten, B.J.; Lauhon, L.J.; Hersam, M.C., All-Printed

Ultrahigh-Responsivity MoS_2 Nanosheet Photodetectors Enabled by Megasonic Exfoliation. *Adv Mater* **2022**, *34*, (34).

S3. Noureen, S.; Rehman, S.U.; Batool, S.M.; Ali, J.; Zhang, Q.; Batool, S.S.; Wang, Y.; Li, C., Tailoring Bi_2Se_3 Topological Insulator for Visible-NIR Photodetectors with Schottky Contacts Using Liquid Phase Exfoliation. *Acs Appl Mater Inter* **2024**, *16*, (6), 8158-8168.

S4. Gao, L.; Chen, H.; Wang, R.; Wei, S.; Kuklin, A.V.; Mei, S.; Zhang, F.; Zhang, Y.; Jiang, X.; Luo, Z.; Xu, S.; Zhang, H.; Ågren, H., Ultra-Small 2D PbS Nanoplatelets: Liquid-Phase Exfoliation and Emerging Applications for Photo-Electrochemical Photodetectors. *Small* **2021**, *17*, (5).

S5. Kim, J.; Kim, S.; Cho, Y.S.; Choi, M.; Jung, S.; Cho, J.H.; Whang, D.; Kang, J., Solution-Processed MoS_2 Film with Functional Interfaces Via Precursor-Assisted Chemical Welding. *Acs Appl Mater Inter* **2021**, *13*, (10), 12221-12229.

S6. Pulikodan, V.K.; Muhammed, R.; Joseph, A.; Alexander, A.; Anitha, B.; Namboothiry, M.A.G., Flexible Thin-Film Photodetectors Based on Solution-Processed Molybdenum Disulphide Nanosheets. *J Mater Res* **2022**, *37*, (6), 1246-1255.

S7. Sharma, R.; Kumar, A.; Kumari, R.; Garg, P.; Umapathy, G.; Laisharm, R.; Ojha, S.; Srivastava, R.; Sinha, O.P., A Facile Liquid-Phase, Solvent-Dependent Exfoliation of Large Scale MoS_2 Nanosheets and Study of their Photoconductive Behaviour for UV-Photodetector Application. *Chemistryselect* **2021**, *6*, (41), 11285-11292.
